# HIV prevention in favour of the choice-disabled in southern Africa: study protocol for a randomised controlled trial

**DOI:** 10.1186/1745-6215-14-274

**Published:** 2013-08-29

**Authors:** Neil Andersson, Anne Cockcroft, Lehana Thabane, Nobantu Marokoane, Ditiro Laetsang, Mokgweetsi Masisi

**Affiliations:** 1 Centro de Investigación de Enfermedades Tropicales (CIET), Universidad Autónoma de Guerrero, Calle Pino, El Roble, Acapulco, Mexico; 2 CIET Trust Botswana, P.O. Box 1240, Gaborone, Botswana; 3 McMaster University, Hamilton, ON, Canada; 4 CIET Trust, 71 Oxford Road, Johannesburg 2196, South Africa; 5 Minister for Presidential Affairs and Public Administration, Office of the President, Private Bag 001, Gaborone, Botswana

**Keywords:** HIV prevention, Gender violence, Southern Africa, Cluster randomised controlled trial

## Abstract

**Background:**

Most HIV prevention strategies assume beneficiaries can act on their prevention decisions. But some people are unable to do so. They are ‘choice-disabled’. Economic and educational interventions can reduce sexual violence, but there is less evidence that they can reduce HIV. There is little research on complex interventions in HIV prevention, yet all countries in southern Africa implement combination prevention programmes.

**Methods/Design:**

The primary objective is to reduce HIV infections among women aged 15 to 29 years. Secondary objectives are reduction in gender violence and improvement in HIV-related knowledge, attitudes and practices among youth aged 15 to 29 years.

A random sample of 77 census enumeration areas in three countries (Botswana, Namibia and Swaziland) was allocated randomly to three interventions, alone or in combination, in a factorial design stratified by country, HIV rates (above or below average for country), and urban/rural location. A baseline survey of youth aged 15 to 29 years provided cluster specific rates of HIV. All clusters continue existing prevention efforts and have a baseline and follow-up survey. Cluster is the unit of allocation, intervention and analysis, using generalised estimating equations, on an intention-to-treat basis.

One intervention discusses evidence about choice disability with local HIV prevention services, to help them to serve the choice-disabled. Another discusses an eight-episode audio-docudrama with community groups, of all ages and both sexes, to generate endogenous strategies to reduce gender violence and develop an enabling environment. A third supports groups of women aged 18 to 25 years to build self-esteem and life skills and to set up small enterprises to generate income.

A survey in all clusters after 3 years will measure outcomes, with interviewers unaware of group assignment of the clusters. The primary outcome is HIV infection in women aged 15 to 29 years. Secondary outcomes in youth aged 15 to 29 years are gender violence and protective knowledge, attitudes, subjective norms, intention to change, agency, discussion of prevention and practices related to HIV and gender violence.

**Trial registration:**

Trial registration number: ISRCTN28557578

## Background

Apart from post-exposure prophylaxis for reported rape, current HIV prevention strategies all address people who can implement their prevention decisions: the choice-enabled [1]. Promoting abstinence [2], male or female condom use [3,4], microbicides [5] or reduced concurrency [6,7] does not take account of people without power to implement their prevention choices. These include victims of gender violence, and many involved in transactional and trans-generational sex. For these choice-disabled, interventions requiring safer choices [8] have muted relevance.

The scale of choice-disability in southern Africa is not trivial. One in every 10 school children in South Africa is sexually abused every year [9]. One in six women in the region experiences physical intimate partner violence each year [10]. Far from the image of a fully empowered professional sex worker insisting that clients use condoms, transactional sex is very widespread in southern Africa and characterised by power inequalities that disable prevention choices. For children who have to trade sex to get through school, for young people to get or to keep employment, or for single mothers who provide for their families through sexual favours, negotiating power is seriously limited by power differentials [11-13].

Another dimension of choice-disability is alcohol use, which temporarily reduces the ability to implement prevention choices [14-16]. Among the HIV-positive, alcohol is associated with delays in seeking treatment [17] and difficulties with HIV medication compliance [18].

One effect of choice-disability is that many people cannot insist on condom use. The paradox of forced passivity is that this disenfranchised group lands up driving the epidemic. In addition to their own risk, inability of the choice-disabled to protect themselves increases the risk of their offspring and of everyone who has sexual contact with them.

Sexual violence means more people at HIV risk through sexual trauma (tears and abrasions), but violence also affects the victims’ world view and way of life [19]. It can lead to rationalisations that make non-consensual sex seem normal or at least expected - a culture of sexual violence. Sexual abuse also affects the way survivors interpret efforts to reduce their risks [20]. Victims of childhood abuse and repeated sexual assault are at greater risk of adopting unsafe sexual behaviours that further increase exposure to HIV infection [21]. Our 2002 school survey found South African children who had experienced forced sex were more likely to say they would spread HIV intentionally [9].

A systematic review of gender violence and HIV [22] stressed the indirect effects of violence on choice disability. The review concluded that victims of childhood sexual abuse are more likely to be HIV-positive, and to have high risk behaviours. Perpetrators of gender violence are themselves at increased risk of HIV infection, as their victims have often been victimised before and have a high risk of infection. Including perpetrators and victims, at least one-third of the southern African population is involved in the gender violence-HIV dynamic.

Two randomised controlled trials from South Africa are relevant. A trial of a structural intervention (income enhancement and gender training) in Limpopo province showed 55% reduction on inter-partner violence. In the time allowed and including many older women, it did not change HIV rates [23]. The other trial reported a non-significant reduction in HIV as the result of a learning/motivational programme [24]. Neither trial focused on the key age group of incident HIV cases (women aged 15 to 29 years).

### Primary objective

In Botswana, Namibia and Swaziland, to reduce the rate of HIV infection within 3 years among women aged 15 to 29 years living in communities receiving three community-based interventions, alone or in combination, focusing on people who are not able to implement choices to protect themselves against HIV infection, compared with young women living in communities without the interventions.

### Secondary objectives

To reduce gender violence (intimate partner violence and sexual violence) among women aged 15 to 29 years living in intervention communities; to improve protective knowledge, attitudes, subjective norms, intention to change, agency, discussion of prevention, and practices related to HIV and gender violence among women and men aged 15 to 29 years living in intervention communities; to measure whether there are synergies (interaction effects) between the three interventions; to assess the resource implications of the community based interventions.

## Methods

### Study population

The trial involves 77 census enumeration areas (EA). The included EAs (the clusters) are those previously selected as a population-weighted random sample in three countries (Botswana, Namibia and Swaziland), stratified by urban/rural location and region [10]. There are 25 EAs each in Botswana and Swaziland and 27 in Namibia. All people living in intervention clusters are eligible to participate in the interventions within that cluster, with specific targeting according to the specific intervention (see below). The impact (on the primary and secondary outcomes) is measured among women and men aged 15 to 29 years living in the intervention and control clusters, contacted in a house-to-house survey of contiguous households from a random starting point and including some 100 respondents in each cluster.

### The interventions

#### Concerting prevention in favour of the choice-disabled

To concert (verb) is to arrange by mutual agreement; from the French *concerter*, to bring into agreement. A service support intervention concerts existing prevention initiatives and services in favour of the choice-disabled; those less able to implement HIV prevention choices [1]. The intention is to increase effectiveness of local efforts without added public investment. In each intervention community, field workers identify a local contact (such as a school teacher, health worker or local entrepreneur) to promote HIV prevention in favour of the choice-disabled. They also identify local prevention players (church leaders, health workers, private practitioners, teachers, traditional leaders), and review access for the choice-disabled, emphasising the likely benefits if services could be concerted in favour of the choice-disabled. As well as working with individual service providers, the field workers and local contact try to develop partnerships between different providers. In around one-half of the concerting sites, a traditional healer focused on spiritual dimensions of choice disability and the four population groups involved (young and old women, young and old men). A small fund supports development of functional partnerships and activities they undertake to raise the profile of choice disability within the community.

#### Enabling a favourable community environment through primary prevention education

In 2002 a large national survey of youth in South Africa led to an audio-drama called Beyond Victims and Villains (BVV) [25]. Updated with results of 2007 surveys in Botswana [26], Namibia [27] and Swaziland [28], this eight-episode audio-drama brings the research results into community-wide structured discussions, aiming to generate local initiatives to reduce gender violence. Discussion of each episode ends by asking what the community can do about it and action planning for local strategies to reduce gender violence. Participants include youth and elders groups, church groups, sports groups, community forums, leaders and elected representatives, schools-based groups, community theatre groups, and community-based organisations. The intervention is open to all: men and women, younger and older members of the community.

#### Structural intervention focused on young women

The Focused Workshop (FW) combines advice and training for young women, helping them to take advantage of existing government and non-government offers, with support to increase self-esteem and assertiveness. The intervention targets young women in the community, particularly those not in school or employment. Health workers and others help to identify those they believe can particularly benefit from the intervention. This begins with 1 week of training, covering skills of communication, negotiation, resisting peer pressure, and building assertiveness and self-esteem. The participants discuss their local economy and choose an income generating enterprise they might develop together. Follow-up supports the young women to develop their enterprise and to apply for loans and grants and provides additional specific training as necessary.

The intervention does not provide capital or loans, but discusses with young women what each can bring to the project, in cash or in kind. The intended empowerment dynamic is ‘self-capitalisation’ of the enterprises, which might involve bringing knowledge (for example, how to make soap or floor polish), labour and small contributions of cash. The project matches the funds raised by the young women when this can make a quantum difference to reach a functional goal. Additional sessions for the groups, as appropriate, cover how to work in a team, conflict resolution, funding and earning opportunities, and how to access them, and practical skills such as preparing a résumé or business plan, and budgeting. Some groups hold a community feast to consolidate their training in budgeting, logistics and delivery. At this feast they present their enterprise idea to their community, including community leaders and invited people such as those from relevant government departments. Organising this feast consolidates their training and changes their standing in the eyes of their community, and in their own eyes.

### Control group

The control group consists of those clusters assigned to receive only existing government and other prevention programmes, which also continue in all intervention clusters.

### Primary and secondary outcome measures

The primary outcome is reduced HIV infection in women aged 15 to 29 years. We will measure this using micro-ELISA of eluted dried blood samples (DBS) from finger-prick blood samples. Fieldworkers collect the finger-prick blood spots on standard filter-paper cards, each spot completely filling a printed circle on the card. The theoretical risk of exposure while taking the DBS sample is virtually eliminated by using single-use safety lancets with a blade that retracts immediately into the hub after use. DBS samples are stable at ambient temperatures, easy to handle and transport, and analysed to the same extent as serum or plasma samples [29]. A small study in India compared ELISA HIV testing on DBS samples with ELISA testing with paired venous blood samples, and reported sensitivity of 100% and specificity of 100% [30]. A South African government laboratory in Johannesburg will analyse the DBS in an anonymous fashion (results will not be returned to individuals - see Ethical considerations). Further testing of samples positive for HIV antibodies will identify recent infections and allow estimation of incidence of new HIV infections. A separate assay detects the presence of anti-retroviral therapy, allowing correction of results from the recent infection tests.

To link the sample HIV result with individual risk factors from questionnaire responses, we use adhesive labels printed with a unique barcode, fixed to the sample, the completed questionnaire and an administrative sheet.

Secondary outcomes, measured among men and women aged 15 to 29 years, include:

a) Choice-disability as measured by intention and sense of agency to insist on the use of condoms; reliance on or disempowerment by transactional sex; sex when inebriated; sense of agency, especially related to multiple concurrent partners;

b) Rates of intimate partner violence and sexual violence.

We will also examine:

c) Combined (interaction) effects on HIV risk and other outcomes of the three interventions; and

d) Costs of HIV cases avoided by the interventions.

We will estimate gender violence as the proportion of respondents reporting physical intimate partner violence or sexual violence in the last year. We will also measure other prevention dynamics, like age of sexual début, condom use and number of concurrent partners. These indicators have high levels of reliability and validity, and are used in DHS and other large surveys in the region.

### Timing

The duration of the intervention is 3 years. We hypothesize that this will be long enough to allow the interventions to have an effect on behaviours, and hence HIV risk, within the communities as a whole (not just those individuals directly involved in the interventions). We will use a reference period for measured behaviours and experiences of 1 year and the recent infection HIV virology testing reflects new infections within a period of <1 year.

### Assignment

**Figure 1:**
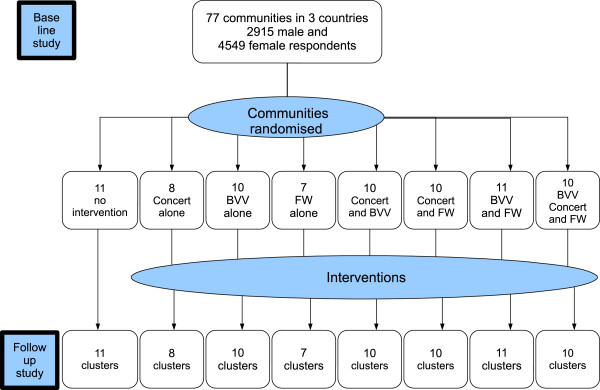
Participant flow, numbers and timing of randomisation assignment, interventions and measurements for each cluster.

Figure 1 shows the participant flow, numbers and timing of randomisation assignment, interventions and measurements for each group. Cluster is the unit of randomisation, intervention and analysis.

#### Generation of allocation schedule

We used a random sample of EAs in each country, drawn for a previous survey [10], based on the latest national census in each country, stratified by urban/rural location. A control and three intervention arms (see Figure 1, factorial design) [31] allows each intervention to be tested on its own and in combination with others.

#### Allocation concealment and timing of assignment

Centralised randomisation (using http://www.random.org/) was undertaken by an epidemiologist not involved in the fieldwork, following the baseline survey. The randomisation was not blocked, but similarly sized clusters were stratified by country, urban/rural and by HIV level at baseline. Prevention activities are obvious to residents in the intervention clusters, although partnerships will be very local, for example, between the local teacher, nurse and traditional healer. Some secondary outcomes (particularly conscious knowledge) could be influenced by knowledge of intervention status. Other indicators like a reduction in gender violence or decisions about multiple partners would be less susceptible to this bias.

The survey manager for the follow-up (DM) is not aware of the exposure status of the communities. Interviewers in the follow-up survey are unaware if they are interviewing in intervention or control clusters. Data managers and operators are kept unaware of intervention status of clusters. The principal analysis will be blind of exposure status, using only group labels for each cluster.

### Sample size and justification for assumptions underlying the power calculations

The original sample size calculations prior to the baseline survey used population-weighted UNAIDS reported HIV prevalence estimates of 34% in women aged 20 to 24 years across Botswana, Namibia and Swaziland, extrapolated to women aged 15 to 29 years [32]. To detect a 30% reduction in HIV prevalence (from 34% to 26%) in any one comparison, the trial required 38 clusters in each arm, with 27 women in each cluster. The factorial design provided this. Figure 1 shows a total of 38 clusters receiving Concerting, 41 receiving BVV, and 38 receiving FW.

Since the baseline study, new virological testing procedures have become available to measure recent HIV infection cases. Among women in the baseline study, HIV-positive rates increased steadily year by year from 2.8% at the age of 15 years to 47.7% at the age of 29 years, an average increase of 3% per year of life. We used this as likely annual incidence in the control group of clusters, revising the power calculations in the light of the baseline findings. Over 3 years, we would expect 9% of women in this age group to become newly HIV-positive. Assuming a range of 0% to 18% (k=0.5) in control communities, follow-up of 65 women in each of 38 clusters would detect a 37% reduction in incidence (9% to 5.7%) with 80% power at a significance level of 5%. These calculations assume no interaction effects and a two-sided test with cluster as unit of primary analysis. Calculations used a spreadsheet based on the trial simulator devised by Taylor and colleagues [33].

Secondary outcomes (gender violence, and related knowledge and attitudes) are more common than HIV infection. It is possible that reduced gender violence may reduce HIV rates directly, but it could also increase the proportion of the population enabled to benefit from all other prevention interventions, increasing their impact correspondingly. As the sample size estimate refers to the primary outcomes, the trial may be inadequately powered for some economic comparisons.

### The proposed analysis

#### General

The analysis and reporting will follow the CONSORT guidelines [34] adopting an intention-to-treat principle for all outcomes, such that all clusters are analysed as assigned, even if some of those assigned to receive one or more interventions failed to implement the interventions fully. We will summarise results of cluster demographics and baseline outcome variables (both primary and secondary) using descriptive measures: mean (standard deviation) or range (minimum-maximum) for continuous variables and number (percent) for categorical variables. We will use multiple imputation to handle missing data [35]. All statistical tests will be two-sided at the 0.05 level of significance. The Bonferroni method will adjust the level of significance for testing for secondary outcomes to keep the overall level at alpha=0.05.

Results will be expressed as effect (or odds ratio/relative risk reduction for binary outcomes), corresponding two-sided 95% confidence intervals and associated *P* values. *P* values will be reported to three decimal places with values less than 0.001 reported as <0.001. Adjusted analyses will be performed using regression techniques to investigate the residual impact of key baseline characteristics on the outcomes. Goodness-of-fit will be assessed by examining the residuals for model assumptions and chi-squared test of goodness-of-fit. Analyses will rely on CIETmap [36] which includes a windows interface with the popular statistical programming language R.

#### Primary and secondary analyses

There is no clear-cut best practice for analysis of a cluster randomised controlled trial with factorial design [37]. We propose analysis using GEE to incorporate the factorial nature of the design and differences at baseline [38], assuming an exchangeable correlation structure within clusters.

#### Supplemental analyses

Sensitivity analyses will examine correlated outcomes using multivariate techniques; and serial correlation of outcomes within clusters over time. A single impact analysis is proposed after the follow-up survey in the fourth year.

Planned subgroup analyses focus on subgroups of young women [39]. Age is a core issue in gender violence and HIV incidence. A history of sexual abuse is likely to affect responses across a range of themes. Use of alcohol and other substances may be informative. Engagement with HIV prevention and treatment services is also gender stratified, with knowledge, attitudes and uptake of prevention options highly gendered. The subgroup analysis will include interaction terms of the subgroup variable with the intervention variables in the model. A further subgroup analysis will examine the effect of the presence of other programmes for HIV prevention, youth empowerment and reduction of gender violence active in the clusters, with this information collected at the time of the impact survey.

### Missing data

#### Response bias

We expect under-reporting of gender violence in administered questionnaires. This may differ in BVV intervention clusters, as the increased dialogue about choice-disability, gender violence and HIV risk might increase disclosure in the intervention sites. This bias would reduce the measured impact of the intervention. Crucial to understanding this will be the comparison between different intervention sites: there is no reason to expect an increase in disclosure with the Concert or FW interventions.

Around 20% of people contacted in the baseline did not want to give a finger-prick blood sample; we did not proceed with the interview in these cases. Response rates to individual questions varied and non-response to some questions was as high as 6%. Self-selection (decision not to participate or not to answer certain questions) is a concern, given the personal nature of some questions. Those who opt not to respond may be most at risk; but these non-response biases should be similar for intervention and control clusters. Participation in the interventions is more difficult to predict. We will use an intention-to-treat analysis, assuming anyone in the cluster had the exposure directly or indirectly.

### Ethical considerations

The survey and finger-prick blood sample do not present more than minimal risk to participants [40]. There is a theoretical risk that disclosure about gender violence can precipitate painful memories. Another risk is that the interventions actually miss the choice-disabled. The recruitment to interventions might inadvertently exclude those who are excluded from most community benefits. We implemented each intervention first in a pilot site in Botswana to generate experience in this respect, allowing us to adjust our approach to increase participation of the choice-disabled.

The trial received approval in Botswana by the Health Research and Development Committee, Ministry of Health (PPME-13/18/1 Vol IV(4), 26 August 2008), in Namibia by the Ministry of Health and Social Services (17/3/3/AP, 22 July 2008), and in Swaziland by the Scientific and Ethics Committee, Ministry of Health and Social Welfare (MH/599B, 26 August 2008).

1. Individual informed consent: Using a standard script, interviewers explain to respondents the nature of the survey and its voluntary nature. They explain that participants may decline to answer any questions and may terminate the interview at any time. They ask respondents for their consent to participate, including giving a finger-prick blood sample, and participants sign to indicate their consent. For participants under the normal age of consent a parent or guardian of the young person signs to indicate their consent.

Community interventions: Prior to randomisation, in each community we identified a suitable community leader able to speak on behalf of the community. We explained the possible interventions and that they might or might not be allocated to receive an intervention, and obtained their permission to include their community in the trial.

2. Samples: Field workers do not proceed with the interview for any potential participant not wishing to give a finger-prick blood sample. They refer anyone requesting the result of the test to the local voluntary counselling and testing (VCT) service for a test, having previously notified the centre about the survey. There is no provision to provide results to participants, as there is no way to link named individuals to the samples, and no provision for counselling. The samples will not be used for any other purpose.

3. Ensuring confidentiality: Training of field workers and data operators emphasises their responsibility for maintaining confidentiality of all information they have access to during the work. We will report only grouped findings, in a way that does not allow identification of any individuals or individual communities. The questionnaires will bear no individual names or identifying marks.

4. Protection of emotional wellbeing: Some of the questions might awaken distressing memories. The interviewers are not professional counsellors but have been trained in how to handle situations when a respondent discloses sensitive information. We provide participants (in the surveys and interventions) with contact information for local services where they can seek help, where these exist.

5. Security of data: Digital records are kept securely and accessible only to the project leader and the lead statistician and their designated assistants. Data entry operators are not allowed to remove data from its centralised secure environment. Original paper records are securely transported, stored, retained and finally destroyed in accordance with our guidelines for security, storage and eventual destruction of paper records.

## Discussion

This factorial cluster randomised controlled trial anticipates different mechanisms from three interventions: one on the enabling environment, one on the services, and one on empowering young women. Gender violence, distorted traditionalist views of gender hierarchy, gendered economic dependence and substance or alcohol abuse all lead to choice disability in HIV/AIDS prevention. Enabling choice shifts power balances and reduces the disdain for the safety of others that is often part of sexual violence and transactional sex. Perhaps most importantly, by increasing the number who can choose existing prevention actions - abstention, condom use or reduced concurrency - purposive enabling of choice will multiply the impact of existing HIV prevention strategies. If more were able to opt for abstention, condom use or reduced concurrency, this would most likely have a measurable impact.

### Limitations

Particularly in Swaziland, a small country with good communication between communities, there could conceivably be some contamination between clusters. This is less likely in Botswana and Namibia, where large distances separate sites.

The sample in the baseline and follow-up surveys represents only those present in the households when the interviewers visit. Young men and women in the target age group may be absent due to work outside the cluster or not near their homes. This could bias the sample towards those without remunerated employment, who could also be those with lower levels of education. This bias would be consistent across intervention and control clusters.

### Focus on the vulnerable

Very few governments in southern Africa address the needs of those who have no agency to implement health choices or to access health services when they need them [39]. This project makes vulnerable people the centre of health services research which, whatever the result, could be relevant to health policy and systems research in many other settings [41]. In addition to specific information on the three preventive interventions, the trial tests combinations of interventions in a factorial design. The research examines a short-term structural intervention to change the ecology of transactional sex for the choice-disabled [42].

## Trial status

As is common in a cluster randomised controlled trial, all clusters were randomised at the beginning of the trial, immediately after the baseline survey. Analysis of the impact assessment is pending, scheduled for 2013.

## Abbreviations

BVV: *Beyond Victims and Villains* audio-drama; DBS: Dried blood spot; ELISA: Enzyme-linked immunosorbent assay; FW: Focused workshop; GEE: Generalised estimating equation; HIV: Human immunodeficiency virus; RCT: Randomised controlled trial.

## Competing interests

The authors declare that they have no competing interests.

## Authors’ contributions

NA conceived of and designed the trial and drafted the manuscript; AC helped to design the trial, leads implementation, and helped to draft the manuscript; LT participated in design and contributed to the manuscript; NM, DL and MM participated in design and implementation, and contributed to the manuscript. All authors read and approved the final manuscript.
